# Karla Soares-Weiser: curating evidence to support knowledge translation

**DOI:** 10.2471/BLT.20.030320

**Published:** 2020-03-01

## Abstract

Karla Soares-Weiser talks to Gary Humphreys about her experiences in clinical practice, medical research, and systematic reviewing and her vision for a better Cochrane Library.

Q: How did you become interested in public health?

A: I started to see that health was more than just about health care or medicine when I was a medical student and was sent to do a six-month internship in a rural area in the final year of my studies. Coming from medical school, I had been very focused on the diagnosis and treatment of disease, but in Minas Gerais, the state I was sent to, I learned a lot about what we now refer to as the social determinants of health. I had grown up in a remote fishing village myself and knew about the conditions in which poor people lived, but Minas Gerais, and particularly the city of Itaipe, was quite an eye opener for me. I remember a child who came to me with a big mass on his neck. It looked like a cancerous growth, but turned out to be a rare form of tuberculosis known as mycobacterial cervical lymphadenitis. Of course, this was devastating for the child and I really wanted to help him, so I decided to visit his home to talk to his family. I will never forget it. He was living in one room with maybe twenty other people. Half of them were infected with tuberculosis. The social services people had very limited resources, but were able to get the family into decent accommodation and the child got better. It really brought home to me how important determinants like poverty and unsanitary living conditions can be.

Q: You went on from medical school to become a psychiatrist. What inspired that change of direction?

A: In my paediatric work I was constantly presented with children who had mental health issues and I came to the view that I could give more support by taking care of their minds instead of their bodies. That led me to an interest in the psychosocial stresses children are subjected to and eventually to doing a master’s in mental health epidemiology. My thesis was focused on a survey of psychiatric conditions in five Brazilian cities done in the 1980s. The survey had a huge impact on my thinking. It was the first time I really appreciated how prevalent mental health problems are, but also the importance of the socio-economic factors driving them, especially racial discrimination.

Q: You started working with the Cochrane Collaboration very early in your career. How did that come about?

A: When I finished my master’s degree, I got a scholarship to do a doctorate at the Federal University of São Paolo. It was my supervisor who made me aware of Cochrane. He came back from the second Cochrane Colloquium at McMaster University in Canada and said, “We may be able to get you a scholarship to work with these people at Oxford University in the UK (United Kingdom of Great Britain and Northern Ireland)”. It was somewhere I had always dreamed of going. Also, I thought that working with the Cochrane systematic review process would dovetail perfectly with my doctorate, which was on evidence-based health care. So I applied for the scholarship, having scrambled to learn English in the meantime, and 6 months later I was in Oxford, working with an incredible, idealistic group of people, not just learning about the systematic review process, but also doing reviews myself. I published 9 Cochrane reviews during my two and a half years there.

“60% of Cochrane Library content is already free to all under our open access policy.”

Q: I understand you got into the business of reviewing research on a freelance basis after your doctorate.

A: That’s right. Essentially because I got married and had three children! I also moved to Israel. Being freelance gave me the flexibility I needed to be able to experience motherhood while also pursuing my career. The youngest of my three boys was born in 2003 and it was in that year that I began working as a systematic reviewer at large for different users of research.

Q: Did working directly with research users change your perspective on systematic reviews?

A: Yes, especially when I started working with policy makers, which was something I did through the company I created in 2009. I worked with the UK and Norwegian governments, the World Health Organization (WHO), and the universities of Nottingham and Liverpool among others. I remember going to present the results of a vaccine review to a group of policy makers from Africa at WHO. In the middle of the presentation, I realized that I had lost their attention. So, I stopped the presentation and asked them what the problem was. They said, “We can see that your methodology is sound, but we really need information that we can understand and act on”. That was an important moment of realization for me. It made me realize how important it is to communicate your findings in a way that allows them to be used.

Q: In 2015 you decided to close your company and join the Cochrane Collaboration full time. What was behind that decision?

A: It was partly a realization that I had reached a point with my company that required even greater commitment than I was already making. Also, Dr David Tovey, the editor-in-chief at that time, invited me to come in as deputy editor-in-chief. He said he thought there was a lot we could do together to support the Cochrane community and achieve Cochrane’s goals. I was excited by the prospect of joining the organization with which I had worked for so long and which had become so big. I led the development of the change management plans for the Cochrane Review Groups, which included the creation of eight thematic networks. I also initiated the development of Cochrane Response, which is based on the company that I had before and caters to stakeholders who sometimes need results faster. Cochrane Response works in collaboration with Cochrane groups to meet that need using a team of professional systematic reviewers.

*Q: You became the new Editor-in-Chief of the Cochrane Library in June 2019, taking over from* *Dr. Tovey. What are you currently focused on?*

A: My broad remit is to ensure continuity and realize Cochrane’s vision of improved health, informed by high-quality, relevant and up-to-date synthesized evidence. More specifically, I am focused on improving access to Cochrane studies.

Q: Does that mean we can hope for full open access to Cochrane studies in the future?

A: This year we are planning to consult with external and internal stakeholders to achieve a common understanding of the challenges, barriers and opportunities for Cochrane in delivering universal, immediate open access to Cochrane Reviews, whilst continuing to ensure financial sustainability for the organization. However, it is important to remember that 60% of Cochrane Library content is already free to all under our open access policy, while institutions in countries in Groups A and B of the WHO’s HINARI Programme have free access to all of our content. We also have national licenses in more than a dozen countries.

“We put together a new conflict-of-interest policy that will […] come into force this year.”

Q: What about translation into other languages?

A: We now produce the Cochrane Library in English and Spanish and translate both the abstract and the plain language summary of reviews in another 16 languages. Views of our website have more than tripled in the past three years, at least in part because of our increased availability in languages other than English. In October 2019, the governing board approved a strategy to increase multi-language dissemination and we are working on how to support groups in the production of better plain language summaries of our reviews. We are also working in collaboration with organizations, such as WHO and Wikipedia to make our content more accessible.

Q: I understand you have also been working on a new conflict-of-interest policy?

A: That’s right. Cochrane has always been committed to independence and transparency, but we felt that that there was more we could do to minimize bias. It is for this reason that we put together a new conflict-of-interest policy that will be published soon and will come into force this year.

Q: Can you describe the key elements?

A: The basic idea is to make Cochrane systematic reviews completely free of commercial influence, and to minimize other conflicts of interest. The proportion of conflict-free authors in a team will increase from a simple majority to 66% or more. Authors of industry-funded clinical studies eligible for inclusion in a Cochrane review will be prohibited from being the first or last author on that review. Reviews funded by not-for-profit organizations with a specific interest in the outcome will be assessed by Cochrane’s conflict of interest arbiter panel. Cochrane authors will also have to declare non-financial interests and think critically about how these might influence the results of the review.

Q: How will you implement the policy?

A: We are appointing a dedicated team that will include a research integrity editor who will be an expert in the conflict of interest field. We will also make changes in our editorial management system to enable improved monitoring of reporting of conflicts of interest throughout the life cycle of reviews.

Q: It sounds like you have a busy year ahead of you.

A: That’s right, but I have always been a hard worker, and together with the worldwide Cochrane community I am dedicated to carrying this great collaboration forward.

